# Somnambulism

**Published:** 1894-06

**Authors:** J. Michell Clarke

**Affiliations:** Physician to the Bristol General Hospital; Lecturer on Practical Physiology in University College, Bristol


THE BRISTOL

### Medico-Chirurgical Journal.

JUNE, 1894.

### SOMNAMBULISM.

BY

J. MICHELL CLARKE, M.A., M.D. Cantab., M.R.C.P.,

*Physician to the Bristol General Hospital; Lecturer on Practical Physiology in University College, Bristol.*

In sound sleep, all the functions of the brain and spinal cord except those connected with the maintenance of life itself are in abeyance. The order in which the various functions are lost is: (1) loss of the higher mental faculties, (2) of the motor functions, relaxation of muscles, and (3) loss of common sensation and of the special senses. They are awakened in the reverse order: the sensory faculties may be readily aroused into activity in light and fairly easily even in sound sleep. This is no doubt a remnant of the ancestral condition in which man had at all times to be on the alert against hostile attacks.

When sleep is disturbed by dreams, the course of events is as follows: The dream may be caused by some stimulus extrinsic to the brain: for instance, a loud noise may be the starting point for a long series of exciting events, a neuralgic pain suggest the stab of an enemy's knife, or a fluttering of the heart from imperfect digestion of a hearty supper a fall from a precipice (other instances will occur to everyone); or, on the other hand, a dream may arise in processes of ideation started in the brain itself. In dreaming, a long series of illusions is gone through—figures and scenes pass before the eyes, imaginary sounds are heard, conversations carried on, speeches made and actions performed, but here the process stops; although a long train of associated images of memory may be aroused, no muscular or other action follows. Sensation, or rather ideation, gives rise to illusion, but illusion does not result in action. Occasionally the illusion of a simple dream may lead to action, but when a movement takes place the dream comes to an end and the process is abruptly interrupted by the sleeper awaking.

In the most vivid dream I have had of late years, I thought the candle was setting fire to the bed-curtains, and awoke to find myself sitting up in bed, blowing vigorously and shouting "Blow it out!" In the state intermediate between sleeping and waking, hallucinations are prone to occur, but, as in dreams, are rarely followed by action—or if movement does result it puts an end to them; and in some conditions of extreme fatigue there is a state in which the mind is active, sensitive to impressions from without, or in other words able to appreciate all that is going on around it, but there is a feeling as if it were separated from or had lost control over the limbs, which are in a state of general relaxation.

If, without awaking the dreamer, the motor centres are put in action by the illusion of the dream, the condition of somnambulism is arrived at. An exceptionally vivid dream is the motive-power which gives rise to hallucination, and this in its turn prompts to action: a sensory image of memory gives rise to an illusion, this illusion is acted upon, and the process goes a step further than in dreaming and calls into play the motor centres. Somnambulistic actions are of all grades, from the simple to the most complex. Note first that the sleep-walker's energies are entirely absorbed and dominated by an extremely vivid illusion, under the influence of which his actions are concentrated, to the exclusion of all other objects, upon the accomplishment of some deed suggested by it. His attention being thus wholly taken up, he is like a man in ordinary life who is engaged in some absorbing pursuit which renders him oblivious to all else that is going on around him.

The well-known instance of J. S. Mill, who composed his *System of Logic* during his daily walk from Kensington to the India House, passing along the crowded streets with an air of total pre-occupation but never colliding with the lamp-posts or with other walkers, is a case in point. Similarly, a soldier may not know he has been wounded until after the excitement of the fight is over. The somnambulist presents the same state in a still more perfect form; all his faculties are more completely concentrated on one object than in any other mental condition with which we are acquainted. Because, however, the somnambulist may be touched, spoken to, and even carried to bed, without perceiving it, we must not jump to the conclusion that touch and the other special senses are for the time in abeyance. In most somnambulists the visual and tactile senses at any rate seem to be preternaturally acute; but all the senses are directed only to one special aim, and are acute only when used to this end. The field of perception as a whole is narrowed, but in one particular direction is exalted: it loses in range, but gains in intensity in that sphere in which it is retained. In short, the sleep-walker is "possessed" by one idea dominating all his actions. I shall only briefly allude to the actions carried out during sleep-walking, as everyone is more or less familiar with them. A sleep-walker may get out of bed, walk on the parapets of houses or along narrow ledges in situations in which in his ordinary state he would become giddy and fall. He may come to a river and swim across it, avoid obstacles and dangers, and pass through the most difficult places in safety: the concentration of the mind upon the desired object is so complete as to inhibit the feelings of timidity or terror which under ordinary conditions would arise and paralyse his will-power.

He may not only feel no fear, but also fail to recognise that certain actions are impossible, and so sometimes walk out of a window and be dashed to pieces, or, being unable to swim, be drowned in the stream into which he has passed. Such accidents would presumably be most likely to occur in imperfect states of somnambulism, when the perceptive centres are not fully aroused, and in which the sleep-walker walks along with his eyes closed.

The actions performed are often so complex and so difficult of accomplishment, and the component muscular movements so suitably co-ordinated and adjusted thereto, that one must conclude that their successive stages are as a rule realised by the sleep-walker. Sensations must reach the highest cerebral centres and be perceived; the manner in which they are acted upon presupposes judgment and even intelligent choice.

A further point is that the same person in the somnambulistic state almost invariably performs the same actions. A certain automatic regularity attends all the movements—so much so, that an observer who has frequently watched the sleep-walker can predict exactly what the latter will do next, and the stage at which he will return to bed. Shakspere well knew this, and also the complicated actions that may be gone through, when he makes the attendant say of Lady Macbeth: "I have seen her rise from her sleep, throw her night-gown upon her, unlock her closet, take forth paper, fold it, write upon it, read it, afterwards seal it and return to bed,—yet all this while in a most fast sleep." Another instance is that of a woman who invariably gained the roof of the house, walked along the parapet of that and the adjoining houses, carrying her pillow in her arms and holding it as if it were a baby. Another woman always got up and prepared a poisonous potion by putting matches in a glass of water; and so on.[1]

It is interesting to note in connection with this repetition of the phenomena of the attack that simple dreams frequently recur in the same way, and are identical in all their details. No doubt the sameness of the somnambulistic acts is to be accounted for by the recurrence of one particular dream.

Personally, I have two dreams which recur at intervals of one or more years. In one, I am walking about in a town full of the ruins of the most beautiful Gothic churches; in the other, I am in a place which remotely resembles Lynmouth and Lynton. The streets and buildings in these dream-places are always exactly the same, so that I seem to be walking about in a place quite familiar to me and in which I have no difficulty in finding my way. I have never been able to recall any place that I have

[1] *Dictionary of Psychological Medicine*, 1892, Art. "Somnambulism."

actually seen that exactly corresponds to these dream-places, but they may be memories of photographs or pictures.

As to the various functions of the nervous system in the somnambulist, the eyes may be open[1] or closed, generally the former, and the sense of vision as a rule seems to be acute. Hearing may be absent or present. Touch is generally preternaturally acute, but is said to be sometimes absent, so that the somnambulist has been carried to bed without awaking. As indicated above, I doubt whether this proves the sense of touch to be absent, but would rather consider that just as the mind is exclusively bent on achieving the action prompted by the illusion and is oblivious of all beside, so the special senses are acute in aiding the carrying on of any action which leads to the desired end—*e.g.* in avoiding obstacles, etc.; but at the same time, there may be no perception of any sensory stimuli outside this.

There is no doubt of the activity of the intellectual processes in somnambulism. It has been proved that children have learned their lessons whilst in the somnambulistic state and repeated them correctly the next day. Speaking, singing, and playing on musical instruments may be successfully accomplished.

The only constant and essential sign of somnambulism is the forgetfulness of all that has passed during the period of somnambulism, however complicated the actions may have been.[2] The vivid dream, however, which preceded the somnambulistic state may sometimes be recalled on waking, and then connected with the particular deed done during sleep.

Now, we have to meet the very interesting question as to whether the somnambulist's actions take place without his being conscious of them, without his knowledge,—are, so to say, more or less automatic; or whether he is at the time fully conscious of his own actions, although not altogether of his surroundings, but afterwards forgets all that has occurred in

[1] Thus Shakspere makes the doctor say: "You see her eyes are open;" and Lady Macbeth's maid reply: "Ay, but their sense is shut."

[2] See Janet, "Quelques définitions récentes de l'Hystérie," in *Arch. de Neurol.*, vols. xxv., xxvi., 1893; and Azam, *Hypnotisme et Double Conscience.* 1893, p. 151.

the somnambulistic state. The answer to this question I shall defer for a few moments.

A further point about somnambulism is, that there is good evidence that in a return of the somnambulistic condition the memory of the events that took place during previous attacks is most often preserved, although completely lost in the waking state. This is analogous to the condition in the "recurrent dreams" mentioned above, and is also found in what is called induced somnambulism—that is, a condition essentially resembling sleep-walking in its main features which can be evoked in a suitable subject by hypnotic suggestion (mesmeric trance). I cannot enter upon the subject of hypnotism, but note in passing that in induced or hypnotic somnambulism all the faculties of the mind are wholly under the control of an outside influence—that of the operator; whilst in spontaneous somnambulism they are under the domination of an intrinsic influence—that of the vivid hallucination or dream. This I take to be the main point of difference between the two states. In induced as in spontaneous somnambulism, after the desired action or speech has been accomplished all further memory of them is lost; but when the subject is subsequently hypnotised and again enters the somnambulistic state, he has a more or less complete remembrance of all the events of the former somnambulism, but of none of those of his normal life. He may thus have two existences—the first or normal, and the second or somnambulistic one,—each with its special memory for what takes place in its own recurrent phases, and absolute want of memory for the events of the other state.

Dr. Azam[1] traces stages between this secondary condition and ordinary sleep. Thus, he says, if a mother speaks gently and in a monotonous tone of voice to a child of eight or ten years, wrapped in the sound slumber of that age, the child does not awake, but answers. One can often direct his thought at will and get the child to say what in his waking state he would not have revealed, cause him to turn in his bed, drink, &c. In some persons this condition is still better developed; thus, there is the well-known case of the

[1] *Op. cit.*, pp. 151–153.

naval officer whose friends were able to suggest dreams to him, and who, sleeping on a bench, precipitated himself on his head on the ground, under the idea that he was diving into the sea to save a friend from drowning. A still further development of this secondary state is reached in the many cases now authenticated of persons who pass spontaneously, or at any rate without any cause that can be recognised, into a condition lasting for a variable length of time, in which their mental and moral characteristics and consequent actions become changed; after a time they again return to their normal mental condition. These cases have been rather absurdly called cases of "dual personality"—of "double consciousness." Stevenson in his *Dr. Jekyll and Mr. Hyde* has made use of this curious state in a very striking way.

We might if we pleased speak in the same way of a person subject to ordinary attacks of sleep-walking as possessing two existences or personalities, the abnormal one being of short duration as compared with well-marked cases of "double consciousness." In both cases there occurs an interference with the normal processes of association in the brain during the attacks, by which certain nervous processes are isolated from the rest, but closely bound up with each other, sometimes in an abnormal or unaccustomed connection. Further, these are the only processes of cerebration carried out whilst the condition lasts. But because, on account of interference with the ordinary processes of association, certain mental processes, habits, thoughts, feelings, and the like, are more closely associated with each other than usual, and at the same time isolated from other similar mental processes with which they would as a rule be connected, we can hardly correctly speak of them as constituting a new or secondary personality, and therefore the expression "dual personality," implying as it does that two persons exist side by side in the same individuality, is misleading, unless we carefully keep in mind what is really meant by it. The phenomenon is rather one of inhibition of particular mental activities with persistence of others, and is based on lapses of memory.

These cases of double consciousness differ from those of the hypnotised subject in whom alterations of the personality can be produced by suggestion, in that they occur spontaneously, or rather without our being able to ascertain the cause. Sometimes we can ascertain it: thus, one man used to dream periodically that he must go at once into a neighbouring town, and next morning, after carefully searching through the places where his wife was accustomed to conceal her money, he went off, abandoned his family, indulged in a bout of drinking, and generally ended by recovering from his abnormal state to find himself in gaol. In this case the influence of the illusion of his dream went further than it does in somnambulism and affected his waking life. This is analogous to the cases of persistence of a suggestion made during hypnosis into the post-hypnotic state.

One of the most celebrated cases of double consciousness is that of Félida X. At the age of 14 this woman began to pass into a secondary state characterised by a change in her disposition and character. During the secondary state she remembered the first state, but on emerging from it into the first state she remembered nothing of the second. At the age of 44, this secondary state, superior in quality to the first, had become more frequent and prolonged, and occupied most of her life. During it she remembers most of the events of her past life, but when she returns into the first she is completely oblivious of all that has passed during the secondary state; and this is often very distressing, as when the transition took place in a carriage on her way to a funeral she could not remember which of her friends was dead. She actually became pregnant during one of her early secondary states, and on recurring to the first had no knowledge of how it had come to pass.[1] Many other instances will doubtless be familiar to the reader. The difference between the mental, moral, and often physical characteristics of the two alternating states is generally in such cases marked enough for the relations or intimate friends to be able to recognise in which condition the patient is.

There are allied states, however, perhaps most often connected with epilepsy, which still more nearly approach the

1 This case will be found in full in Dr. Azam's book quoted above, and there is an abstract of it with critical remarks in Prof. James's *Principles of Psychology*, vol. i., pp. 380 and 384, where also other similar cases are given.

condition of spontaneous somnambulism. Persons pass without warning into a secondary state of which they have no memory afterwards, and of whose existence they would have no idea were it not for the gaps thus caused in their lives of which they can give no account. This condition is spoken of as "ambulatory automatism," and it cannot be recognised by any external manifestations, as the presence of the secondary condition in cases of secondary consciousness can, *i.e.* by changes in the character, disposition, or mental condition of the person affected. For in ambulatory automatism the minor daily actions of life are carried on as usual, and there is nothing to betray the existence of an abnormal state. I will quote one instance from Charcot:[1] An agent (A. B.) for a firm in Paris who was accustomed to take round curios for sale, arrived at a client's house in a street in Paris, in the course of his daily round, at 8 p.m. on 18th January. As he did not return to his carriage after some time, the coachman on inquiry finding that he had left the house, returned to the office and advised the principals of their agent's disappearance. From January 18—26 there is a complete gap in A. B.'s memory, but on the 26th he found himself on a bridge in a city strange to him, whilst a military band was passing at the time which might have awakened him. Not liking, for obvious reasons, to ask the name of the town, he inquired his way to the railway-station, and there saw that he was at Brest, a place in which he had never been before, and with which he had no business relations. He found that his clothes were clean and well-kept, his shoes not dirty or worn, so that he could not have travelled the 200 miles on foot, and his general condition was such that he must have had proper food and accommodation during the time; he also discovered that he had spent about £8 in the interval. Much disturbed in mind, and fearing the condition might return, he foolishly confided his story to a gendarme, who promptly concluded he was a clerk absconding with his master's money, remarked that "it was a very fine story" and took him to the lock-up. The man had had other attacks of the same kind, and similar cases are recorded. There was

[1] For full account see *Leçons du Mardi à la Salpêtrière*, 1890, pp. 303 et seq.

absolutely no reason to doubt the man's *bona fides*. Now, the eight days had completely dropped out of the man's life, he had absolutely no memory of them, and yet during all this time we know from his condition that he must have taken food and drink, have slept, have obtained shelter, have paid for all these things, have taken his railway-ticket, and all the time have conducted himself like an ordinary person; at any rate, if he had done anything extraordinary he would certainly have returned to his normal state to find himself in the hands of the police or in a lunatic asylum. The man was perfectly sane and intelligent, and there was no evidence of brain disease.

When we consider the many complicated mental processes which must go on to enable A. B. to take a long railway journey of this kind, to carry out at least the ordinary daily actions of life in an ordinary way without exciting remark, we must conclude that whilst in this state he was in possession of his ordinary faculties, and that all his actions were attended by consciousness. It is impossible to understand how it could be otherwise, how consciousness could be absent under such circumstances, and we can only conclude that there was a complete loss of memory for the events of this second state when he returned to his normal condition. If, however, he could have been seen and conversed with during a recurrence of this secondary condition he might have remembered all the events of former secondary states, and been wholly oblivious of those of his ordinary life.

A similar instance came under my own observation, the cause being probably a slight blow on the head; injuries to the head of all degrees of severity are known to produce this condition, without there being any reason to suppose that any structural damage to the brain occurs. A youth of 18 asked leave from his master one afternoon to return home as he felt indisposed, and it then came out that he had a complete lapse of memory for all that had occurred since the morning. From after breakfast to after lunch his memory was a complete blank, except that he remembered eating some apple-pie. He conducted himself during the morning quite as usual, except that he asked one or two rather trivial questions. He told his comrades on arriving at the office that he had slipped in the snow, and fallen five or six times on his way down. He gave two messages quite correctly to his master, who came in after him. There was no sign of injury or contusion, and his clothes showed no evidence of a fall. He rode a part of the way to the office in a 'bus, and went into the restaurant to which he was accustomed to go, selected and ordered his dinner and chose his seat. In hailing the omnibus and leaving it at the proper place, in getting his dinner at a crowded restaurant, and in doing his office work as usual, he must have gone through a number of complicated actions involving the exercise of all his mental faculties, and have done all this in such a way as not to excite remark or to lead to any suspicion that he was otherwise than in his usual condition.

The condition in these cases is closely allied to somnambulism, and I think affords us an answer to the question I raised earlier—namely, that a person during sleep-walking is conscious, can see, hear, and feel, and form more or less accurate judgments from his perceptions as to the actions best adapted for attaining the end he has in view, but on awaking completely forgets all that has passed during the somnambulism; and although the sphere of this consciousness in the somnambulistic state is limited, there is no ground for the assumption that the actions are sub-conscious, and take place without bringing into activity the higher cerebral centres.

To discuss very briefly the physiology of these allied states. Taking the three grades of nerve centres as—(1) spinal, for simple reflex actions; (2) basal, for more complex reflex activities carried on without consciousness—*e.g.* respiration, deglutition, regulation of heart-beats,—and, according to some, for such activities as speech, swimming, and walking, after these actions have been thoroughly acquired. On this view, they must generally be started on each occasion by a voluntary impulse from (3) the highest cortical centres in the brain, the actions of which are as a rule attended by consciousness. I say as a rule because, although all activities, mental or muscular, which are either novel or require delicately adjusted co-ordinated movements are attended by consciousness, yet actions which have become very familiar to us through frequent repetition, though they demand consciousness at first, can after a time be carried on without it. In striking out fresh paths in the brain or those which are seldom used nervous impulses experience greater resistance, meet with more friction; and it seems to be this resistance which is especially attended with consciousness. On the other hand, in passing along well-worn paths impulses meet with little resistance, and may at last take place without or with a very limited measure of consciousness. Thus, on this supposition the more complex automatic or reflex activities are carried out by the higher (cortical) centres. Now, in sound sleep the activity of all these centres is in abeyance. In dreaming the higher cerebral centres are only in action; though sensations of vision, of pain, occur in dreams, the lower sensory centres may not be aroused, but the images of memory of former sights, pains, smells, &c., deposited in the cells of the highest centres are called up. In somnambulism both the lower and the highest sensory and motor centres are called into activity, but only in a limited way, with strict reference to the illusion which is being acted upon. The rest of the brain lies dormant. Similarly, in induced somnambulism (hypnotism) the operator can put into action certain centres exclusive of all others by means of a potent suggestion. These centres enter into new and as a rule unaccustomed association with each other, and are also isolated in their action from all other centres to an extent that never takes place in normal life, and their excessive activity inhibits the action of other parts of the brain. In both cases the normal processes of association of the ordinary life being for the duration of the somnambulistic state set aside, there is nothing to call up the memory of what has passed until the subject again falls or is thrown into the same condition, when the processes of association for the special events of the somnambulistic or hypnotic state evoke a partial or complete memory of the previous or of all former somnambulisms.

The phenomena of secondary consciousness can be explained in a similar way, the secondary states being prolonged conditions of somnambulism which arise spontaneously in the waking state, and the events of which are associated together in the brain, but dissociated from all other cerebral processes.

